# Functional Feed for Tilapia: Exploring the Benefits of *Aspalathus linearis* Tea Extract

**DOI:** 10.3390/biology14070778

**Published:** 2025-06-27

**Authors:** Grace Okuthe, Bongile Bhomela, Noluyolo Vundisa

**Affiliations:** Freshwater Laboratory, Department of Biological and Environmental Sciences, Walter Sisulu University, Mthatha 5117, South Africa; bbhomela@wsu.ac.za (B.B.); nvundisa@wsu.ac.za (N.V.)

**Keywords:** rooibos tea extract, Mozambique tilapia, functional ingredient, aquaculture feed, growth performance, feed efficiency

## Abstract

To address the growing global demand for aquatic protein and the need for sustainable aquaculture, this study explored *Aspalathus linearis* tea extract as a novel feed additive for *Oreochromis mossambicus* larvae. Over an eight-week feeding trial, the efficacy of diets supplemented with 30% fermented or green rooibos extract was assessed against a control. Both fermented and green rooibos treatments significantly (*p* < 0.05) enhanced larval growth, evidenced by improved weight gain and feed conversion ratios (fermented: 1.50 ± 0.25; green: 1.41 ± 0.07). Notably, *A. linearis* extracts also demonstrated genoprotective potential, as indicated by a marked reduction in micronucleus frequency, most likely attributed to their abundant phenolic compounds. These findings demonstrate that rooibos extract, especially the green variety, can improve growth performance and feed utilization, and also provide genoprotective benefits. The superior outcomes in growth and feed conversion are likely due to the bioactive phenolic compounds, which may enhance palatability, gut health, and nutrient absorption, rather than macronutrient content. This positions rooibos extract as a promising natural functional additive for aquafeed, offering a sustainable strategy to enhance tilapia farming productivity and resource utilization. However, further research is necessary to uncover specific molecular mechanisms, conduct in-depth analyses of gut health and immune responses, and evaluate effects on product quality to facilitate its sustainable and effective integration into aquaculture practices, thereby contributing to both fish health and food security.

## Simple Summary

This study investigated the application of rooibos (*Aspalathus linearis*) tea extract as a sustainable feed additive for Mozambique tilapia larvae over an eight-week feeding experiment. Diets fortified with 30% fermented and green rooibos extract significantly enhanced larval growth compared to the control group. While the control showed a higher condition factor, the growth efficiency with rooibos extracts was substantial. The findings demonstrate that rooibos tea extract, in both forms, can significantly improve tilapia growth and feed efficiency, presenting it as a promising, cost-effective, and sustainable ingredient for aquafeed for tilapia farming. These findings offer a viable strategy to boost tilapia farming productivity, promoting efficient resource utilization and sustainable aquatic protein production.

## 1. Introduction

The aquaculture sector is undergoing a transformative shift, driven by the escalating global demand for seafood and the imperative of developing sustainable production practices [1]. This has resulted in the aquaculture industry emerging as a pivotal player in global food security, necessitating a comprehensive re-evaluation of traditional feeding strategies [2]. Conventional aquafeeds, heavily reliant on fishmeal and other marine-derived components, present considerable ecological and economic challenges [3]. This dependence not only depletes wild fish stocks but also introduces sustainability concerns related to sourcing, transportation, and processing [4,5]. As commercial fish production expands, the strain on ocean-derived protein sources intensifies, further underscoring the need for alternative, sustainable feed ingredients to sustain aquaculture production [6].

The aquaculture industry’s move towards increased energy-dense diets and streamlined production cycles has amplified the significance of feed composition, influencing growth rates, disease resistance, and overall fish health [7,8,9]. Consequently, there is an exigent demand for functional feeds that transcend basic nutritional requirements, actively promoting fish health, resilience, and environmental sustainability. The integration of functional components like probiotics, prebiotics, and synbiotics offers potential pathways for fortifying aquafeeds, addressing ecological concerns linked to conventional feed formulations, and enhancing disease resistance [10]. The development of such feeds is crucial for the continued growth and sustainability of the aquaculture industry, aligning production practices with ecological well-being and long-term resource management [11].

Recent research underscores the escalating importance of functional feeds in addressing the multifaceted challenges confronting modern aquaculture, including disease management and environmental sustainability. As aquaculture intensifies to meet rising global demand, the sector faces heightened risks of disease outbreaks and environmental degradation, prompting a shift towards innovative dietary strategies [12]. The incorporation of medicinal herbs such as Garlic (*Allium sativum*), Ginger *(Zingiber officinale*) and Moringa (*Moringa oleifera*), for example, into finfish diets has garnered attention for their potential to boost immune responses and disease resistance, presenting a natural alternative to chemical treatments [13,14,15,16,17]. This approach aligns with the growing consumer preference for sustainably produced, chemical-free seafood, driving research into the efficacy and safety of herbal feed additives [18]. The exploration of enzymatic fish protein hydrolysates derived from fish processing waste represents another promising avenue for sustainable aquafeed production. These hydrolysates, rich in proteins, amino acids, peptides, and antioxidants, have demonstrated the potential to enhance growth, feed utilization, immune function, and disease resistance in fish [19]. This utilization of fish processing by-products not only reduces waste but also diminishes the reliance on traditional fishmeal sources, contributing to a more circular and sustainable aquaculture system. However, this practice also raises concerns about antibiotics from fish waste processing in feed. Moreover, the strategic use of feed additives, such as plants, herbs, probiotics, prebiotics, and synbiotics, is gaining momentum as a means to modulate the gut microbiome of farmed fish, thereby promoting host health and improving nutrient utilization [20]. Such methodologies are essential for enhancing disease resistance [21]. The application of prebiotics in aquaculture, for example, is considered an effective solution to improve feed conversion efficiency and promote fish growth [22]. This comprehensive approach, integrating sustainable ingredients and health-promoting additives, signifies a paradigm shift in aquafeed formulation, aiming to optimize fish health, minimize environmental impact, and ensure the long-term viability of aquaculture operations [23].

The imperative for responsible aquaculture practices is further highlighted by concerns surrounding antimicrobial resistance. The utilization of antimicrobials in aquaculture, while sometimes necessary for disease control, can contribute to the development and spread of antimicrobial-resistant bacteria, posing risks to both aquatic ecosystems and human health [24,25,26,27]. Concerns about antimicrobial resistance have also resulted in criticism from multiple stakeholders [28].

Alternatives to antibiotic use, such as vaccination, bacteriophages, quorum quenching, probiotics, chicken egg yolk antibodies, and medicinal plant derivatives, are now being explored [29]. Consequently, contemporary aquaculture nutrition research increasingly focuses on formulating effective feed substitution strategies utilizing components and supplements of plant origin [30,31].

The development and application of functional feeds in aquaculture signify a crucial move toward sustainable and environmentally conscious practices [32]. The exploration of immunostimulatory feed additives has shown promise in enhancing disease resistance and overall health. These additives have demonstrated the potential to stimulate the immune system of aquatic animals, improving their ability to combat pathogens and reducing their reliance on antibiotics. The demand for environmentally friendly aquaculture, coupled with increasing pressure from customers, has triggered better management practices and technical innovation [33]. By applying functional feed additives in animal feeding, one can achieve higher daily growth, better fodder use, higher resistance to diseases, improvements in animal product quality, and a reduction in the harmful effects of animal droppings on the environment [34]. This can be achieved by dietary supplements with herbs.

Beyond their nutritional contributions, various plants and their extracts possess bioactive compounds traditionally employed in animal nutrition as appetite stimulants, palatability enhancers, digestive aids, physiological modulators, and antioxidants, with the potential to prevent or mitigate specific pathological conditions and promote the growth performance. Various plants and their extracts possess bioactive compounds [35,36,37,38]. The empirical evidence supporting the benefits of phytochemicals and herbal products in enhancing productivity within the aquaculture sector is growing [39,40]. Phytochemicals, a broad class of secondary metabolites, are ubiquitously distributed in fruits, vegetables, and plant-derived beverages, including wine and tea [41,42,43,44].

Consequently, tea polyphenols are attracting considerable interest as natural functional ingredients to improve aquatic animal welfare. Research reviews indicate that phenolic acids can enhance growth, feed utilization, immune responses, and disease resistance through their antioxidant activity and the modulation of biological pathways [45]. Their adoption provides an environmentally sound alternative to synthetic additives, offering a vital opportunity to boost aquaculture productivity and contribute to food security. Complementing this, a separate study revealed that dietary tea polyphenols significantly improve the quality of farmed grass carp [46]. This includes enhancements in muscle flavor, an increase in beneficial “healthcare components,” and improvements in physicochemical properties, such as texture. Importantly, the study elucidated the mechanism by which tea polyphenols influence collagen synthesis, thereby directly impacting muscle structure. Collectively, these findings underscore a promising sustainable strategy for the advancing aquaculture sector to produce higher-value, healthier fish, aligning with global food security goals and consumer demand for premium food products.

Rooibos tea, derived from the *Aspalathus linearis* plant native to South Africa, presents a compelling case for its inclusion in both human and animal diets due to its rich composition of bioactive compounds. Its global popularity is evident in its export to over 37 countries, with the European Union, Japan, the United Kingdom, and the USA constituting the majority of rooibos tea exports [47]. The unique sensory profile of high-quality rooibos tea, characterized by honey, woody, floral, and caramel notes, alongside a sweet taste, enhances its appeal as a palatable and health-promoting beverage [48]. Unlike traditional teas derived from *Camellia sinensis*, rooibos is naturally caffeine-free and exhibits condensed tannin content, mitigating the common concerns associated with caffeine-induced anxiety, insomnia, and tannin-mediated iron absorption inhibition [49]. The absence of caffeine makes it a suitable alternative for individuals sensitive to stimulants, while the reduced tannin levels ensure better iron bioavailability from dietary sources.

The health-promoting properties of rooibos tea are also largely attributed to its diverse array of antioxidant compounds, including flavonoids, such as aspalathin, nothofagin, orientin, isoorientin, quercetin, rutin, and luteolin [50]. These compounds contribute to the tea’s notable antioxidant activity, which can combat oxidative stress by neutralizing free radicals, thus preventing cellular damage and reducing the risk of chronic diseases [51]. Studies have demonstrated that rooibos extracts and their constituent flavonoids exhibit potent antioxidant and anti-inflammatory effects, protecting against lipid peroxidation, DNA damage, and inflammation-related disorders [52]. Aspalathin, a unique dihydrochalcone glycoside found almost exclusively in rooibos, stands out for its exceptional antioxidant capabilities and potential to modulate glucose metabolism, offering promising avenues for managing type 2 diabetes [53]. Furthermore, rooibos contains various minerals, including iron, potassium, calcium, copper, zinc, magnesium, and fluoride, contributing to overall nutritional value and supporting various physiological functions, improving the immune system and the ability to detoxify and stimulate [54].

The antioxidant activity of rooibos tea further extends to neuroprotection, with evidence suggesting it may protect against age-related neurodegenerative diseases [55]. Emerging research indicates that rooibos tea could have beneficial effects on bone health, potentially increasing bone mineral density and reducing the risk of osteoporosis. Moreover, the antimicrobial properties of rooibos tea have been investigated, demonstrating its ability to inhibit the growth of certain bacteria and fungi, which could contribute to improved gut health and immune function [56].

Furthermore, the potential applications of rooibos tea extend beyond human nutrition, with growing interest in its use as a functional ingredient in animal feed. The inclusion of rooibos in animal diets could offer several advantages, including improved antioxidant potential, enhanced immune function, and reduced inflammation. Studies have explored the effects of rooibos supplementation on livestock, poultry, and aquaculture species, with promising results observed in terms of growth performance, disease resistance, and product quality. For example, the addition of rooibos extracts to poultry feed has been shown to improve growth rates and meat quality, while also reducing the incidence of oxidative stress and inflammation [57].

Despite the wealth of evidence supporting the health benefits of rooibos tea, further research is also necessary to explore the potential of rooibos tea as a functional ingredient in aquafeed, with a focus on optimizing its inclusion and evaluating its long-term effects on fish health and productivity [58]. In aquaculture, rooibos supplementation has the potential to enhance the immune response of fish, making them more resistant to pathogens. These benefits are especially of interest for the aquaculture industry, where the use of antibiotics can have significant detrimental environmental effects. In general, the use of tea is known to be helpful in improving physiological function and antioxidant activity [59].

Depending on processing methods, rooibos tea is commercially available as fermented rooibos tea (FRT), characterized by its reddish-brown hue and distinct phytochemical composition, or unfermented green rooibos tea (GRT), which retains a greenish color [60]. Key compounds identified in FRT include tannins, specific flavonoids (notably aspalathin and aspalalinin), and various phenolic acids [61]. Aspalathin stands out as the primary flavonoid in GRT and is also a significant component of FRT water extracts [62,63,64]. Given its rich phenolic composition and established health benefits, this study aims to evaluate the potential of *A. linearis* tea extract as a functional ingredient in *O. mossambicus* feed, seeking to contribute to the development of sustainable and efficient aquaculture practices.

## 2. Materials and Methods

### 2.1. The Plant Material

Green and fermented rooibos tea (*Aspalathus linearis*) was exclusively sourced as bulk material directly from the manufacturer, Rooibos Ltd. (Clanwilliam, Cape Town, Western Cape Province, South Africa). This direct procurement strategy was implemented to eliminate the risk of contamination or the inclusion of any non-original substances that might be associated with retail distribution, thereby ensuring the integrity and purity of the plant material for subsequent analyses.

### 2.2. Analysis of Plant Metabolites

Secondary plant metabolite analysis was performed at the Central Analytical Facility (CAF), Stellenbosch University, Western Cape, Cape Town, South Africa. The analytical procedures followed the methodologies established by Akinyede et al. [65] and Amelo et al. [66] which are briefly outlined below.

#### 2.2.1. Fatty Acid Methyl Esters (FAMEs)

The sample (ca. 1.00 g) was extracted with 2.5 mL hexane and 1 mL of 20% sulfuric acid in methanol at 80 °C for 1 h. After cooling, 1.5 mL of 20% NaCl solution was added, vortexed, and centrifuged. The 150 µL hexane (top) layer was transferred to a 2 mL vial insert for GC-MS analysis.

#### 2.2.2. Amino Acids

The sample (ca. 50 mg) was hydrolyzed with 5 mL of 6M HCl at 110 °C for 24 h. After cooling, the solution was diluted (1:1) with 70% methanol (*v*/*v*). A 250 µL aliquot was dried under nitrogen, reconstituted, derivatized with 50 µL MTBSTFA and 100 µL acetonitrile at 100 °C for 1 h, cooled, and injected into the GC-MS.

#### 2.2.3. Sugars and Phenolic Acids

For phenolic (25 mg sample) and sugar (100 mg sample) extractions, 250 µL and 1 mL of 70% methanol were added, respectively. Samples were vortexed and extracted at 60 °C for 3–4 h and 250 µL of each extract was dried under nitrogen.

#### 2.2.4. Sugar Derivatization

Sugars were derivatized with 100 µL of 2% methoxyamine in pyridine (40 °C, 2 h), then with 50 µL BSTFA (60 °C, 30 min). Samples were vortexed, transferred to a vial insert, and injected into the GC-MS.

#### 2.2.5. Phenolic Derivatization

Dried phenolics were derivatized at 80 °C for 1 h with 100 µL acetonitrile and 50 µL BSTFA. Samples were transferred to a vial insert and injected into the GC-MS.

### 2.3. Chromatographic Separation

#### 2.3.1. Fatty Acid Methyl Esters (FAMEs)

Fatty Acid Methyl Esters (FAMEs) were separated by gas chromatography–mass spectrometry (GC-MS) using an Agilent 6890N/5975B MSD system equipped with a CTC PAL autosampler (Agilent Technologies, Santa Clara, CA, USA). Separation was performed on a ZB-WAX column (Phenomenex, Singapore) (30 m, 0.25 mm ID, 0.25 µm film), with helium as the carrier gas, at a flow rate of 1 mL/min. The injector temperature was set to 250 °C, and a 1 µL sample was injected in splitless mode. The oven temperature program commenced at 50 °C for 2 min, followed by a ramp at 25 °C/min to 180 °C (held for 5 min). The temperature then increased at 3 °C/min to 250 °C (held for 2 min), with a maximum temperature of 260 °C held for an additional 2 min. The mass selective detector (MSD) operated in full scan mode, ranging from 35 to 500 *m*/*z*. The source and quadrupole temperatures were 230 °C and 150 °C, respectively, while the transfer line was maintained at 250 °C. Electron ionization (EI) was performed at 70 eV.

#### 2.3.2. Sugars

Sugars were separated using an Agilent 6890N/5975 MSD GC-MS system, also equipped with a CTC PAL autosampler. A Zb semi-volatile column (Phenomenex, Singapore) (30 m, 0.25 mm ID, 0.25 µm film) was utilized, with helium serving as the carrier gas at 1 mL/min. The injector temperature was 250 °C, and a 1 µL sample was injected with a 10:1 split ratio. The oven program was initiated at 80 °C for 5 min, followed by an 8 °C/min ramp to 250 °C (held for 1 min). The temperature then increased at 20 °C/min to 320 °C (held for 5 min), with a maximum temperature of 325 °C held for 0.25 min. The MSD operated in full scan mode (40–650 *m*/*z*), with source and quadrupole temperatures set at 240 °C and 150 °C, respectively. The transfer line was 250 °C, and EI was performed at 70 eV.

#### 2.3.3. Amino Acids

Amino acids were separated on an Agilent 6890N/5975 MSD GC-MS system incorporating a CTC PAL autosampler. A Zb semi-volatile column (30 m, 0.25 mm ID, 0.25 µm film) was employed, with helium as the carrier gas at a flow rate of 1 mL/min. The injector temperature was 250 °C, and a 1 µL sample was injected with a 10:1 split. The oven program began at 100 °C for 5 min, followed by a 20 °C/min ramp to 325 °C (held for 4 min), concluding with a 0.25 min hold. The MSD operated in scan/SIM mode, with a scan range of 40–650 *m*/*z*. Source and quadrupole temperatures were 240 °C and 150 °C, respectively, while the transfer line was at 250 °C, and EI was performed at 70 eV.

#### 2.3.4. Phenolic Acids

Phenolic acids were quantified using a GC-MS/MS system (Thermo TSQ 8000 triple quadrupole MS, Thermo Fisher Scientific, San Diego, CA, USA) operating in Selected Reaction Monitoring (SRM) mode. Separation was achieved on an Rxi^®^-5Sil MS w/integra Guard column (Bellefonte, PA, USA) (15 m, 0.25 mm ID, 0.25 µm film). Helium served as the carrier gas at 1 mL/min. The injector temperature was 250 °C, and a 1 µL sample was injected splitless. The oven program commenced at 100 °C for 4 min, followed by a 10 °C/min ramp to 180 °C (held for 2 min), and then a 20 °C/min ramp to 320 °C (held for 5 min). The ionization source was maintained at 250 °C with a 50 µA emission current for argon collision.

### 2.4. Experimental Procedures

#### 2.4.1. Preparation of Rooibos Tea Extracts and Feed

Aqueous extracts of fermented (FRT) and green rooibos tea (GRT) were prepared by adding 300 g of plant material to 1 L of deionized water in separate clean glass beakers. The mixtures were brewed for 30 min and then allowed to cool to room temperature (≈25 °C) before being filtered. The resulting tea extracts were refrigerated at 4 °C until further use. For diet preparation, 7.5 g of dry tilapia feed pellets were mixed with 30 mL of either deionized water (control), FRT extract, or GRT extract to form a ball-shaped dough. Each diet mixture was stored in labeled containers and frozen at −20 °C until feeding. The ingredients and chemical composition of the commercial feed are shown in Table 1.

#### 2.4.2. Experimental Feeding Groups

Three distinct dietary treatments were formulated and administered to the experimental groups (Table 1). The first, designated as the Control (CBD) group, received a commercial basal tilapia diet sourced from Avi Feed Co., Johannesburg, Gauteng Province, South Africa, to which no rooibos tea extract (0.0% *w*/*v*) was added. The second treatment, the FRT Diet, consisted of the same basal diet supplemented with 30% (*w*/*v*) fermented rooibos tea extract. Finally, the GRT Diet represented the third treatment, comprising the basal diet fortified with 30% (*w*/*v*) green rooibos tea extract.

#### 2.4.3. Experimental Setup for Feeding Trial

All experiments were performed at the freshwater biology laboratory at Walter Sisulu University. *O. mossambicus* larvae were sourced from a registered supplier (Aquaculture Innovations, Grahamstown, South Africa).

Larval fish were acclimatized for two weeks before the onset of the experiments and fed a commercial tilapia diet twice daily. Before feeding trials, fish were starved for 24 h, size-sorted by hand, and randomly stocked into each recirculating aquaculture system (RAS). The RAS consisted of a PVC circular cage connected to a biological filter, a water pump, a UV sterilizer, and an electric water-heater. Tanks were filled with de-chlorinated tap water two weeks before fish stocking. The water flow rate into each tank was 600 L/h. Supplemental aeration was provided in each tank for optimal oxygen dissolution using a regenerative blower and a submerged air-diffuser. The water temperature was maintained at 28 ± 1 °C.

Temperature and dissolved oxygen readings were recorded daily using a handheld Hanna portable Model HI198198 dissolved oxygen (DO) meter (Hanna Instruments, Johannesburg, South Africa (78 ± 2% saturation), and pH (6.5 ± 2) was measured weekly using a Freshwater Aquaculture Test Kit (Hanna Instruments, Johannesburg, South Africa). The photoperiod was maintained at 14:10 h (light–dark), and water in each system was topped up weekly. Fish were cultured on three diets: a control, basal commercial tilapia diet (Avi Feed Co., Johannesburg, South Africa)—(CBD) with 0.0% (*w*/*v*) rooibos tea extract inclusion, a basal diet with 30% (*w*/*v*) fermented rooibos tea (FRT) extract inclusion, and a basal diet with 30% (*w*/*v*) green rooibos tea (GRT) extract inclusion. A total of nine hundred fish (initial weight of 0.54 ± 0.08 g and length of 2.22 ± 0.01 cm) were randomly distributed into the RAS in triplicate at a hundred fish per tank. Each diet was assigned to triplicate tanks of fish, and fish were hand-fed to satiation three times daily for eight weeks at 2% body weight. At the end of eight weeks, samples were withdrawn to measure different growth parameters.

#### 2.4.4. Proximate Composition of Diets

A proximate composition of all diets (Table 1) was performed at the Agricultural Research Council (ARC), Irene, Pretoria, South Africa. The proximate composition of both feeds underwent analysis following the established methods by [67], encompassing measurements of moisture, total nitrogen, crude protein, fiber, ash, carbohydrate, lipid, their respective ratios, energy, and protein solubility. Subsequent to this, the feed’s digestible energy was computed, attributing 16.72 kJ/g to protein, 17.62 kJ/g to carbohydrate, and 37.62 kJ/g to lipid, a calculation derived from [68]. Dry matter was determined by drying samples to a consistent weight at 105 °C, while crude protein was ascertained using the Kjeldahl method [69], with its content derived by multiplying nitrogen by 6.25. Crude lipid was quantified via acid hydrolysis utilizing a Sotex System HT 1047 Hydrolyzing Unit (Tecator Application Note 92/87), followed by Soxhlet extraction with a Soxtec system 2055. Gross energy was assessed using an adiabatic bomb calorimeter (Parr Instruments, Moline, IL, USA), and ash content through combustion in a muffle furnace at 550 °C for 16 h. Each of these analyses was conducted in triplicate.

### 2.5. Measurement of Growth Performance Parameters

At the conclusion of the feeding trial, fish were subjected to a 24 h starvation period before harvesting and data collection to ensure empty guts. From each feeding regime, fifteen fish were randomly sampled from their experimental tanks. These fish were anesthetized with MS222 (Sigma Aldrich, St. Louis, MO, USA). Following anesthesia, individual body weights were recorded to the nearest 0.1 g, and fork lengths were measured with an accuracy of 1.0 mm. Initial body weight (IBW) in grams and initial body length (IBL) in centimeters were recorded at the trial’s start. Similarly, final body weight (FBW) in grams and final body length (FBL) in centimeters were measured at termination, with all length and weight measurements consistently following the methodologies of [70,71]. Additionally, of the fifteen fish, six were further processed. Their viscera (intestines and associated fat deposits, excluding liver or gonads) and livers were carefully dissected and weighed individually. These weights were used to calculate the viscerosomatic index (VSI) and hepatosomatic index (HSI). VSI was determined as viscera weight/(body weight − viscera weight) × 100, and HSI as liver weight/(body weight − liver weight) × 100.

Specific growth rate (SGR) was calculated using Equation (1):

SGR (%) = (100 × (FBW − IBW))/(time (d))(1)

The rate of weight gain (WGR) was calculated using Equation (2):

WGR = 100% × ((FBW − IBW))/IBW(2)

The condition factor (CF) was calculated using Equation (3):

CF = 100% × FBW/FBL(3)

The survival rate (SR) was calculated using Equation (4):

SR = 100% × NF/NI(4)

NF is the number of fish at the end of the feeding trial, and NI is the number of fish at the beginning of the feeding trial.

### 2.6. Tissue Fixation and Processing

The method of [72] was adopted. Briefly, liver and spleen samples were fixed in Histochoice fixative (Sigma-Aldrich, St. Louis, MO, USA) overnight at room temperature, before being rinsed in running tap water and then in 70% ethanol. Tissues were preserved in 70% ethanol until further processing. Fixed tissues sufficient for analysis were dehydrated through an ethanol series and cleared in a xylene solution. Tissues were embedded in paraffin wax (Paraplast^®^, Merck, Darmstadt, Germany) at approximately 58 °C in the embedding machine (Thermo Scientific Microm EC 350, Hidalgo, TX, USA). Sections were manually cut to 5–7 μm thickness, using a Leica manual microtome (Leica RM2235: Leica Biosystems, Nussloch, Germany), floated in a water bath at 37 °C, and then positioned on Poly-Prep glass slides (Sigma-Aldrich, St. Louis, MO, USA). Tissue sections were dewaxed in xylene, hydrated in ethanol series [73], stained with Harrison’s modified hematoxylin, counterstained with eosin, and mounted in DPX. Images of stained tissues were examined and captured using a Leica DM 750 fluorescent microscope (Leica Microsystems, GmbH, Nussloch, Germany), attached to a DFX 310 FX digital camera (Leica Microsystems, GmbH, Nussloch, Germany), and analyzed using Leica LAS imaging software version 4.5.

### 2.7. The Micronucleus Assay (MN)

Blood samples were collected from sampled fish by cardiac puncture with a heparinized syringe (needle 0.5 mm thick) for micronucleus analysis. Blood drops from each fish were placed on a Poly-Prep glass slide (Sigma-Aldrich, St. Louis, MO, USA) with the use of a pipette. Glass coverslips were used to spread blood onto slides. Slides were air-dried for 2 h at room temperature and then fixed in methanol for 10 min. For each experimental group, ten slides were prepared. Five slides from each treatment group were stained with a fluorescent dye, acridine orange (AO), to provide information about the physiological or pathological state of fish during the feeding trial. Images of the stained material were viewed and captured using a Leica DM 750 fluorescent microscope (Leica Microsystems, GmbH, Nussloch, Germany). Units similar to and next to the main nucleus were interpreted as micronuclei [74].

### 2.8. Statistical Analysis

#### 2.8.1. Growth Performance Parameters

Statistical Package for Social Sciences (IBM, Armonk, NY, USA, SPSS, V. 5) was used to analyze fish growth data and a one-way analysis of variance (ANOVA) was performed to test the differences between the growth parameter means of different treatments. Duncan’s post hoc test for multiple comparisons was performed to determine significant differences among groups. A significant difference was considered when *p* ≤ 0.05.

#### 2.8.2. Plant Metabolite Analysis

The concentration (mg/L) was measured for each of the five herbal samples (clients) for each of the compounds (IDs). There were three replicates for each measurement. The hypothesis that the vectors of the client means were all equal was to be tested. The multivariate permutations test (PERMANOVA) was performed by using the Adonis function in the vegan package of R (version: 2.7-1).

## 3. Results

### 3.1. Sugars in GRT and FRT

Rooibos tea extracts contain three significant groups of sugars: the monosaccharides (D-glucose and D-fructose), the disaccharides (sucrose and α-lactose), and a trisaccharide (raffinose). The FRT and GRT extracts have the same fruit sugar concentration (sucrose = 90 mg/mL). It is worth mentioning that the FRT extract had a higher glucose concentration (≈78 mg/mL) when compared to the GRT, with 40 mg/mL. On the other hand, the α-lactose concentration was lower in the FRT extract (8 mg/mL) compared to the GRT extract, which contained 28 mg/mL (Table 2).

The amino acid profiles of both fermented rooibos tea (FRT) and green rooibos tea (GRT) exhibited a comprehensive range of essential and non-essential amino acids, as shown in Table 3. Glutamic acid represented the most abundant amino acid in both samples, accounting for 15.14% in FRT and 14.56% in GRT. Leucine was also prevalent, being the second most abundant in FRT at 11.12%, while aspartic acid showed a higher proportion in GRT (13.44%) compared to FRT (11.04%). Other significant amino acids included proline and alanine, with each contributing over 8% to the total amino acid content in both tea types. Conversely, methionine, asparagine, serine, and tyrosine were present in lower concentrations, generally ranging between 2% and 4%. Overall, the two processing methods, fermentation and green processing, yielded largely comparable amino acid compositions, with minor variations observed across individual amino acid percentages.

Both FRT and GRT extracts contain various phenolic acids, namely, 4-hydroxybenzoic acid, vanillic acid, protocatechuic acid, *p*-coumaric acid, syringic acid, ferulic acid and caffeic acid Table 3. The mean concentration of phenolic acid profiles is also shown in Table 3. The results of the PERMANOVA test show that FRT means concentrations were much greater than the corresponding GRT ones.

### 3.2. Growth Response of Larval Fish to FRT and GRT Extracts and Histopathology of Selected Organs

Experimental fish groups fed a diet containing rooibos tea extracts (FRT and GRE) showed an improvement in the growth indices compared to the group fed with the CBD, but with a significant increase in the Viscerosomatic Index (VSI). Fish groups fed GRT extracts had the highest VSI compared to other test groups. Weight gain and the specific growth rate of fish fed with FRT and GRT were also higher than those of the control (those fed the CBD), showing the role natural plant products (metabolites) played in fish growth. The feed conversion ratio (FCR) was higher in fish fed the BCD (2.32 ± 0.57), followed by FRT (1.50 ± 0.25) and GRT (1.41 ± 0.07). Similarly, fish-fed BCD had a higher condition factor (CF): CBD (13.14 ± 4.87), GRT (6.70 ± 2.53), and FRT (6.29 ± 2.45). There was no significant difference between the FRT and GRT treatment groups (ANOVA, *p* ˂ 0.05), Table 4.

#### 3.2.1. Liver and Spleen Histopathology

**Figure 1:**
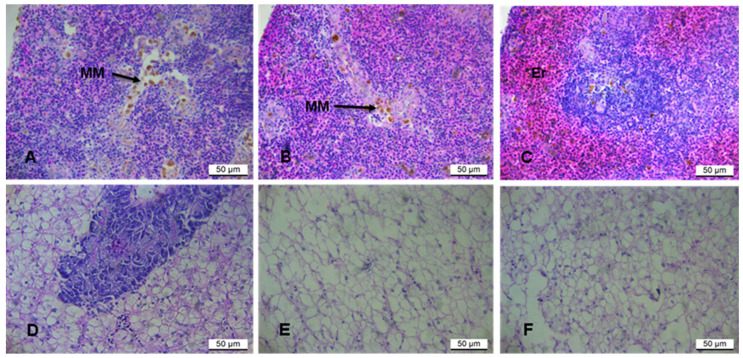
Hematoxylin and eosin (H&E)-stained sections of *O. mossambicus*. Spleen (**A**–**C**); (**A**) CBD = control with melano-macrophages (MM) within the white pulp; (**B**) FRT with fewer melano-macrophages and a few erythrocytes (Er); (**C**) GRT with a distinct boundary between the white and red pulp and a few scattered melano-macrophages within both the red and white pulp. Liver (**D**–**F**); Fat vacuoles and large hepatocytes with displaced nuclei characterized the liver of fish fed FRT (**E**) and GRT extracts (**F**).

The potential effects of rooibos tea extracts on the liver and splenic functions were evaluated. The spleen of fish-fed CBD showed a higher degree of loosely dispersed macrophages, specifically in the white pulp, with a reduced number of blood cells (Figure 1A). At low magnification, the boundary between the white and red pulp was blurry, with the red pulp being more diffuse. The FRT group exhibited a balance of red and white pulp and few blood cells, with a moderate occurrence of scattered macrophages mainly restricted to the white pulp (Figure 1B). The GRT group indicated fewer macrophages within the diffuse white pulp and a prominent red pulp surrounding the white pulp (Figure 1D). The spleen was full of red blood cells and had an apparent boundary between the white and the red pulp. Normal polygonal hepatocytes with normal blood vessels were observed in fish-fed CBD with a moderate degree of cytoplasmic vacuolization, and hepatocytes showed clear boundaries (Figure 1D). Fat vacuoles and large hepatocytes with displaced nuclei characterized the liver of fish fed FRT (E) and GRT extracts (F) (Figure 1E and 1F, respectively).

#### 3.2.2. The Micronucleus (MN) Assay

**Figure 2:**
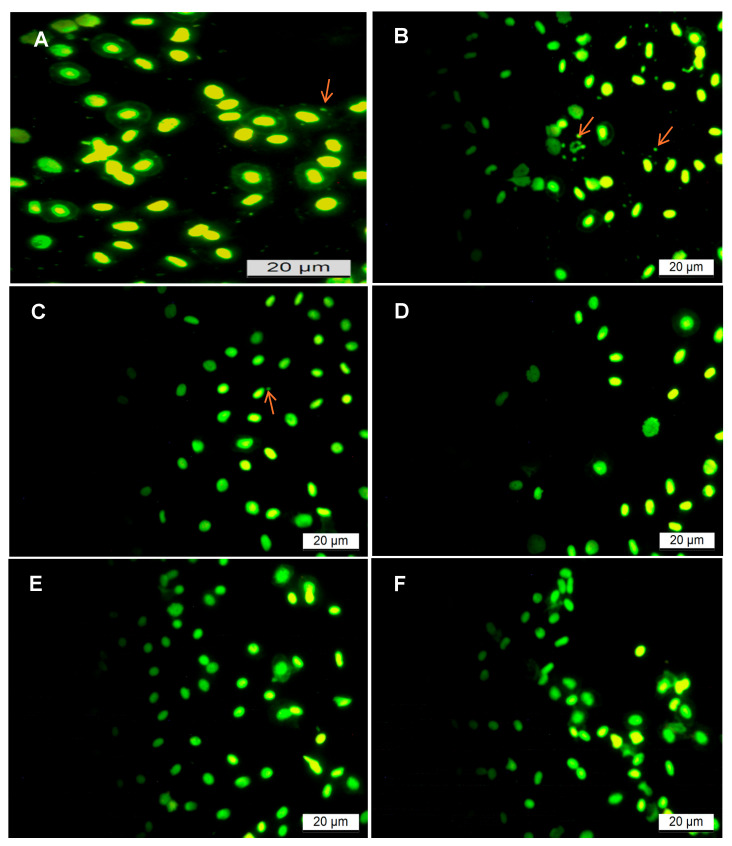
Photomicrographs of the micronucleus (MN) assay in fish fed different rooibos tea extracts for 6 weeks; acridine orange (AO) stain. (**A**,**B**) Diet 1 CBD = (control); (**C**,**D**) Diet 2 (FRT); and (**E**,**F**) Diet 3 (GRT). Arrows in (**A**–**C**) indicate the micronucleus.

The mutagenic potential of the tea extracts was examined using the MN assay in blood samples to evaluate different chromosomal alterations in blood cells. No signs of toxicity, such as loss of appetite, were noted during the feeding trial in all groups. Contrary to the expected results, MN was seen in the control group blood samples and was rarely seen in experimental groups (Figure 2), affirming the absence of cytotoxic effects of the diet. Supplementing the commercial basal diet (CBD) with rooibos tea extracts did not enhance the frequencies of MN frequencies, inferring the non-toxicity of the tea extracts. There were no differences between the GRT and FRT treatment groups. These effects might be related to the natural compounds present in the extracts, which did not interfere with the DNA synthesis process.

## 4. Discussion

This study investigated the effects of fermented (FRT) and green rooibos tea (GRT) extracts as dietary supplements on larval *Oreochromis mossambicus* growth, health, and genotoxicity, addressing the need for sustainable and effective alternatives in aquaculture feed. Feed is a prominent expense in intensive fish farming, and its quality and quantity are crucial for appropriate growth and reproduction [75]. Diet supplementation is a major consideration in intensive aquaculture, particularly during early growth stages. The application of functional plant-origin feed supplements offers safer, sustainable alternatives to antibiotics, enhancing growth performance, improving fish health, and contributing to food security.

### Larval Fish Growth Response to FRT- and GRT-Supplemented Diets

All three experimental diets exhibited very similar proximate compositions, indicating that the inclusion of FRT and GRT extracts did not drastically alter their fundamental nutritional makeup compared to the control diet. These findings suggest that while the extracts did not substantially change overall macronutrient and mineral percentages, they likely introduced other beneficial qualities, such as improved digestibility, altered nutrient bioavailability, or the presence of bioactive compounds. Further studies are needed to understand the specific impact of FRT and GRT on the availability and digestibility of these nutrients, as well as the presence of other beneficial compounds not captured by proximate analysis.

Fish are known to modify feed intake to satisfy their energy demand. In this study, the inclusion of FRT and GRT extracts in the basal diet (CBD) improved feed utilization and growth in larval fish, which may indicate efficient nutrient uptake, resulting in an increased organo-somatic index (VSI%). The GRT-supplemented diet appeared more suitable for growth and feed conversion ratio (FCR) than the FRT diet. This suggests that adding rooibos tea extracts, especially GRT, may influence feeding behavior in larval fish, favoring enhanced growth performance.

Tilapia generally accepts a wide variety of plant-based diets. The observed improvements in growth performance and feed utilization are consistent with previous studies demonstrating the beneficial effects of plant extracts, such as green tea (*Camellia sinensis* L.) [76,77], as well as medicinal and other plant extracts [78,79,80], in various aquaculture species, including tilapia. Such inclusions have been shown to improve feed utilization, nutrient absorption, hematological parameters, and immune response [22,81,82,83]. The significantly improved FCR in the rooibos extract groups (fermented: 1.50 ± 0.25; green: 1.41 ± 0.07) compared to the control group (which had a higher, less efficient FCR) clearly indicates that fish consuming rooibos-supplemented diets were more efficient at converting feed into biomass. This enhanced efficiency is a key indicator of improved digestive health and metabolic performance, likely mediated by the properties of the rooibos extracts. While the rooibos groups showed superior weight gain, the control group exhibited the highest condition factor (K = 13.14 ± 4.87). The condition factor measures a fish’s robustness relative to its length. This observation, while appearing counterintuitive, suggests that while rooibos promoted efficient linear growth and overall biomass accumulation, the control fish might have accumulated more adipose tissue relative to muscle or, conversely, the rooibos extracts encouraged leaner muscle mass gain.

In aquaculture, efficient linear growth and lean muscle deposition are often more desirable than a high condition factor if this implies excessive fat accumulation. This finding highlights the need for further studies on body composition analysis (e.g., lipid content, muscle protein) to fully elucidate the physiological impact of rooibos supplementation on fish morphology. The comparable growth performance between both rooibos extract treatments implies that fermented and green rooibos extracts offer similar benefits regarding weight gain and FCR for larval fish, despite subtle differences in their processing. This indicates that the beneficial components are either robust across both forms or that both forms contain equally effective compounds.

Rooibos’s potential health benefits and bioactivity are linked to its naturally occurring phenolic contents. These high polyphenolic contents confer considerable antioxidant properties, with scavenging effects on free radicals that may prevent cellular oxidative damage [84]. Two classes of phenolic acids, hydroxybenzoic and hydroxycinnamic acids, were found in the analyzed rooibos tea extracts. Protocatechuic acid (PCA) was the most abundant in GRT, followed by ferulic, vanillic, and syringic acids. PCA, a major metabolite of complex polyphenols, has anti-inflammatory properties [85,86] and has been reported to improve growth in freshwater algae and enhance meat quality and body weight in broilers [87]. The authors suggest that PCA may induce an active cellular immune response, leading to good health and a higher body weight, which aligns with the current study’s findings. Therefore, the high content of PCA in GRT extracts explains the superior growth performance and increased body weight observed in fish-fed GRT extracts. Ferulic acid (FA), like PCA, is a secondary metabolite that can enhance growth performance and carcass characteristics in ruminants by reducing lipid peroxidation.

Phenolics are generally regarded as strong natural antioxidants with key roles in various biological and pharmacological properties, such as anti-inflammatory, antimicrobial, antiviral, and signaling molecules [88,89]. In farm animal production, phenolics act as growth-promoters by stimulating digestive enzyme secretions, decreasing pathogenic bacteria in the gastrointestinal tract (GIT), or modulating gut morphology [89]. Studies have shown improved FCR in poultry feed supplemented with natural extracts containing phenolic compounds, associated with altered intestinal surface area and better nutrient absorption [90,91]. Phenolic compounds are also known to balance beneficial and pathogenic bacteria in the GIT, maintaining gut health and enhancing growth [87]. They may also improve feed flavor and palatability, promoting feed intake and growth performance [92]. Despite these benefits, aquafeed rarely exploits phenolic acid-rich natural extracts for the same purpose [88]. Our study findings provide insight into the usefulness of phenolic acid-rich rooibos extracts as a potential aquafeed supplement, which can likely be attributed to their antioxidant potential and ability to avert cell damage from free-radical oxidation reactions.

Although the effects of FRT and GRT extracts on gut health were not evaluated in the current study, the presence of various phenolic compounds in rooibos tea extracts may have improved feed flavor and palatability, leading to better growth performance. Alternatively, the higher growth rate and improved FCR in the GRT treatment group could be due to alterations in the intestinal surface area and digestive enzyme activities, resulting in better food absorption, increased body weight, and less feed wastage. The observed variations in glucose and lactose concentrations for the two rooibos tea extracts could be attributed to the breakdown of α-lactose in the GRT extract upon fermentation, yielding glucose and galactose units. This accounts for the observed increase in glucose concentration in the FRT extract and a consequential decrease in its α-lactose concentration. Sugars, while non-essential dietary nutrients in aquafeed, represent an inexpensive source of valuable dietary energy and contribute to feed pellet integrity by reducing density, increasing stability, and optimizing binding activity during manufacturing.

The hematological parameters of tilapia are critical indicators of their health and physiological status. Studies on various plant extracts, including rooibos, have demonstrated their potential to modulate hematological indices. For instance, dietary supplementation with red seaweed extract (*Gracilariopsis lemaneiformis*) significantly increased hemoglobin levels and improved blood parameters, suggesting that marine algae extracts can enhance blood health in tilapia. Similarly, carob syrup in tilapia diets has improved immune parameters, such as phagocytic activity, without adverse effects on hematological indices [93]. Rooibos tea extract has been associated with a reduction in pro-inflammatory cytokines in human leukocytes, suggesting potential similar effects in fish leukocytes [94]. Furthermore, studies on tilapia have shown that dietary supplements can improve immune responses under stress conditions, which may be relevant when considering rooibos extract [95].

The dietary inclusion of *A. linearis* extract, as demonstrated in this study, can enhance the immune responses of Mozambique tilapia through various mechanisms. This plant extract is rich in bioactive compounds that can stimulate both innate and adaptive immune responses, thereby improving the overall health and resilience of the fish in several ways:

- Enhanced Phagocytic Activity: Rooibos extract can enhance the activity of phagocytes, which are crucial for the innate immune response and pathogen clearance. Dietary supplements like rooibos extract can enhance the phagocytic index in tilapia, similar to xanthones from mangosteen. Various dietary supplements positively influence tilapia immune responses, including phagocyte activity, suggesting rooibos extract’s similar role in enhancing innate immunity [96]. Natural extracts can improve overall health and disease resistance in tilapia, indicating rooibos’s broader potential in aquaculture.
- Modulation of Immunomodulatory Cytokines: The extracts may promote the expression of immunomodulatory cytokines, such as interleukin-1 beta (IL-1β) and interferon-gamma (INF-γ), which are vital for orchestrating immune responses.
- Increased Leukocyte Counts: The inclusion of functional ingredients like rooibos may lead to increased leukocyte counts, which is essential for effective phagocytosis [97].
- Reduction in Oxidative Stress: Compounds like aspalathin and nothofagin in rooibos exhibit strong antioxidant properties, reducing oxidative stress and inflammation in fish, which can enhance immune function [98].
- Improved Antioxidant Enzymes: The extract may elevate levels of antioxidant enzymes, such as catalase and glutathione peroxidase, further supporting immune health.

While the benefits of rooibos extract are promising, its effectiveness as a supplement can vary based on dosage and the specific immune challenges faced by the fish. Further research is needed to fully understand the mechanisms and optimize the use of rooibos in aquafeeds. The incorporation of rooibos tea extract in the diet of Mozambique tilapia has been shown to positively influence leukocyte counts, which are crucial for the immune response. Research indicates that rooibos extract not only enhances growth performance but may also modulate immune parameters, including leukocyte activity.

The genoprotective potential of rooibos tea extracts was clearly demonstrated through the observed reduction in micronucleus (MN) frequency. The MN assay is a highly sensitive and widely accepted biomarker of genotoxicity, providing crucial insights into chromosomal damage or mitotic spindle dysfunction within aquatic organisms. Its global validation by various research institutions underscores its reliability for evaluating environmental samples, natural products, and biological agents. This assay has proven particularly sensitive in detecting DNA damage induced by diverse environmental pollutants, including polycyclic aromatic hydrocarbons (PAHs) and various pesticides [99,100], making it a robust tool for ecotoxicological assessment.

In fish, the MN assay on peripheral erythrocytes is a particularly advantageous and frequently employed method for assessing cytogenetic damage [101,102,103]. This is due to its simplicity and speed, and the fact that erythrocytes are highly exposed to circulating genotoxic agents and undergo division, leading to MN formation. The frequency of micronuclei in fish erythrocytes can be influenced by a range of factors, including diet and water quality [104,105], making it an excellent indicator of the physiological or pathological state of fish during feeding trials.

The low occurrence of micronuclei in the rooibos-supplemented treatment groups, as revealed by acridine orange (AO) staining, can be directly attributed to the potent antioxidant and free radical scavenging properties conferred by the phenolic compounds abundant in rooibos tea extracts [84]. Plants naturally synthesize these bioactive polyphenols as a defense mechanism to protect their vital metabolic functions against the oxidative stress induced by free radicals, enabling their survival in diverse and often highly oxidative environments [106].

Recent advancements in rooibos research further support these findings. *A. linearis*) is uniquely rich in dihydrochalcones, notably aspalathin and nothofagin, which are recognized for their exceptional antioxidant capacity. Aspalathin, in particular, is almost exclusively found in rooibos and has been shown to combat oxidative stress effectively [107]. The genoprotective effects of such natural antioxidants have been extensively documented across various animal models, including laboratory animals and humans.

In the context of aquaculture, maintaining cellular integrity and mitigating oxidative stress is critical for fish health, growth, and overall productivity, especially under intensive farming conditions, which often involve stressors like high stocking densities, fluctuating water quality, and increased metabolic demands. The inclusion of rooibos extracts in the diet likely provides a nutraceutical benefit, strengthening the fish’s endogenous antioxidant defense system. This improved antioxidant status directly contributes to the prevention of cellular oxidative damage, which in turn reduces DNA fragmentation and chromosomal aberrations that lead to micronuclei formation, thereby enhancing fish resilience under intensive farming conditions. This study’s demonstration of reduced MN counts in rooibos-fed *O. mossambicus* is thus consistent with the broader understanding of rooibos’s anti-mutagenic and cytoprotective properties. It highlights the potential of rooibos tea extracts as a functional feed additive in aquaculture, not only for enhancing growth performance and feed efficiency but also for promoting fundamental cellular health and genetic stability in farmed fish, thereby contributing to a more robust and resilient aquaculture systems. Further research is needed to elucidate the specific molecular pathways by which individual rooibos polyphenols exert their genoprotective effects in teleost fish.

In teleost fish, the spleen is recognized as a principal hematopoietic and peripheral lymphoid organ. Its critical functions encompass erythropoiesis, the storage and destruction of aged erythrocytes, and the phagocytosis of cellular debris. It is also a key site for antibody production [108] and plays a pivotal role in antigen presentation and the initiation of adaptive immune responses. Its sensitivity to environmental stressors renders it a valuable biomarker for environmental pollution.

In the current study, the effects of fermented rooibos tea (FRT) and green rooibos tea (GRT) on splenic histopathology were not overtly discernible, with no overt indications of histopathological lesions or degenerative changes observed across experimental groups. However, the detection of a few scattered splenic macrophage aggregates, specifically within the control diet (CBD) group, contrasted with their apparent absence or reduced presence in the rooibos-supplemented groups, suggests a potential modulatory or protective effect of rooibos extracts on splenic immune surveillance and cellular homeostasis. This is further supported by the observed increase in circulating blood cells in the GRT treatment group, which may reflect enhanced hemopoiesis or improved systemic blood flow, contributing to overall physiological vigor. These preliminary observations strongly warrant further investigation using advanced immunohistochemical techniques or flow cytometry to quantify specific immune cell populations and evaluate the expression of immune-related genes, thereby providing a more comprehensive understanding of rooibos’s impact on fish splenic immunology.

The liver, as a central metabolic organ and accessory digestive gland in fish, serves as a highly sensitive and reliable indicator of an organism’s nutritional status and overall physiological health [109,110]. Its histological structure is a primary method for assessing the impact of dietary components on hepatic function, fitness, and production characteristics in aquaculture. Imbalanced nutrient mixtures or anti-nutritional factors in aquafeeds can induce hepatic dysfunction and morphological alterations, including hepatic steatosis, cellular degeneration, and vacuolation of hepatocytes, often indicative of metabolic dysregulation.

In the present study, fish fed experimental diets, particularly those in the FRT treatment group, exhibited discernible morphological alterations in hepatic tissues. These changes included an increased cytoplasm-to-nucleus ratio, nuclear irregularities, and prominent hepatocyte vacuolization. Such findings are consistent with nutritional disorders and metabolic stress previously reported in aquaculture species, thereby corroborating the prevalence of parenchymal changes observed in both the FRT and GRT treatment groups in the current study. Hepatic alterations, such as lipid accumulation, distorted nuclei, and changes in cell membranes, are recognized as key symptoms of liver metabolic burden or toxicity [111,112]. Excessive caloric ingestion can overwhelm the liver’s physiological capacity, leading to lipid accumulation and subsequent cellular stress.

While the definitive extent of liver toxicity directly attributable to FRT and GRT extract inclusion could not be precisely determined in this study, cytoplasmic vacuolization was the most frequently observed hepatocyte alteration. This vacuolar degeneration can arise from a multitude of factors, including lipid droplet accumulation, glycogen storage, or cellular responses to stress, making it challenging to establish a clear threshold between physiological adaptation and pathological states solely based on histological examination. Recent research underscores the complex etiology of hepatic steatosis in farmed fish, linking it to high-fat diets, oxidative stress, and impaired lipid metabolism. Although rooibos extracts possess strong antioxidant properties (as discussed in the micronucleus assay section), their inclusion might have subtly influenced nutrient absorption or metabolic pathways, leading to the observed hepatic responses under specific experimental conditions.

This suggests a complex interaction where beneficial compounds might trigger adaptive metabolic changes, or that the specific dosage/formulation might have led to these observations, even if overall health benefits were observed.

Future investigations should integrate biochemical analyses of liver enzymes, lipid profiles, and molecular markers of oxidative stress and inflammation to provide a more comprehensive understanding of these hepatic changes.

To advance the development of sustainable aquafeeds incorporating Aspalathus linearis extracts, it is imperative that subsequent feeding trials are conducted under diverse and appropriate production conditions. This will enable the precise determination of optimal inclusion levels of rooibos extracts, not only for their beneficial effects on growth performance and overall fish health but also for their influence on crucial product quality attributes, such as filet quality, flavor, and shelf-life, which are paramount for market acceptance.

## 5. Conclusions

Our study focused on the effects of rooibos tea extracts (FRT and GRT) on growth performance in larval *Oreochromis mossambicus*. The findings collectively demonstrate improvements in growth performance, feed utilization, and genoprotective effects, alongside a nuanced impact on organ histopathology. The observed superior weight gain and feed conversion ratios, particularly with green rooibos tea, suggest that the benefits extend beyond basic macronutrient content, and are likely attributable to the bioactive polyphenolic compounds, such as protocatechuic acid, which may enhance palatability, gut health, and nutrient absorption.

The genoprotective effects, evidenced by reduced micronucleus frequency, underscore the antioxidant capabilities of rooibos, contributing to cellular health and genetic stability. While the study highlights promising applications for rooibos extracts in aquafeed, particularly given their local sourcing potential, the observed hepatic alterations necessitate further investigation into optimal inclusion levels and long-term physiological impacts. Future research should focus on elucidating specific molecular mechanisms, conducting comprehensive gut health and immune response analyses, and evaluating effects on product quality. This will ensure the sustainable and effective integration of *Aspalathus linearis* extracts into aquaculture practices, contributing to both fish health and food security.

## Figures and Tables

**Table 1:** Proximate chemical composition (wet basis) of the basal diet.

Diets		CBD	FRT	GRT
Proximate Composition	%			
Dry matter		95.28	95.29	95.32
Moisture		4.72	4.71	4.68
Protein		32.99	32.8	33.17
Fat		4.94	4.75	4.81
Ash		7.03	6.98	7.03
Fiber		3.32	2.69	3.87
Carbohydrates		50.01	50.73	50.31

**Table 2:** Mean sugar concentrations (mg/L) for the herbal compounds.

Client	L_Rhamnose	D_Fructose	D-Glucose	Sucrose	Alpha_Lactose	D_Maltose	Raffinose
SFRT	7.44	1.95	75.01	90.54	6.92	5.58	5.66
SGRT	5.86	21.20	44.25	90.54	27.19	5.85	13.31

**Table 3:** The protein content in the investigated material.

Amino Acid Profile (%)		
Amino Acid	FRT * (%)	GRT * (%)
Alanine	8.62	8.18
Glycine	6.07	5.98
Valine	5.57	5.36
Leucine	11.12	10.99
Isoleucine	4.35	4.22
Proline	9.13	9.42
Methionine	2.41	2.15
Serine	4.05	3.93
Threonine	8.33	7.64
Phenylalanine	6.31	6.22
Aspartic acid	11.04	13.44
Glutamic acid	15.14	14.56
Asparagine	3.89	3.77
Tyrosine	3.97	3.88
Phenolic Acid Profile (μg/L)		
Phenolic Acid	FRT * (%)	GRT * (%)
4-Hydroxybenzoic_acid	183.93	71.72
Vanillic acid	752.47	161.62
Protocatechuic acid	1243	920.2
m-coumaric acid	n/d	n/d
*p*-coumaric acid	129.43	77.75
Syringic acid	1404.98	135.83

* FRT—fermented rooibos tea; * GRT—green rooibos tea; n/d—not detected.

**Table 4:** Growth performance and survival rate of larval Oreochromis mossambicus fed three different diets: commercial basal diet (CBD), and diets supplemented with either fermented (FRT) or green (GRT) rooibos tea extracts.

Growth Parameters	Basal Diet	FRT *	GRT *
Initial number of fish—NI	300	300	300
Initial body weight (g)—IBL	0.55 ± 0.02	0.55 ± 0.02	0.54 ± 0.03
Initial body length (cm)—IBL	2.22 ± 0.41	2.24 ± 0.30	2.21 ± 0.45
Final body weight (cm)—FBW	2.86 ± 0.60 ^a^	4.01 ± 0.88 ^b^	4.13 ± 0.75 ^b^
Final body length (cm)—FBL	5.25 ± 0.51 ^a^	5.51 ± 0.50 ^ab^	5.84 ± 0.46 ^bc^
SGR (%)	3.65 ± 0.40 ^a^	4.38 ± 0.44 ^b^	4.48 ± 0.42 ^b^
Condition factor (%)—CF	13.14 ± 4.87 ^a^	6.92 ± 2.45 ^b^	6.70 ± 2.53 ^b^
Growth (%)	5.15 ± 1.32 ^a^	7.69 ± 1.95 ^b^	7.98 ± 1.65 ^b^
HIS (%)	6.59 ± 1.91	6.75 ± 1.96	7.02 ± 2.10
VSI (%)	21.06 ± 7.10 ^a^	22.91 ± 11.87 ^b^	26.64 ± 12.52 ^c^ *
Rate of weight gain (g)—RWG	2.32 ± 0.59 ^a^	3.46 ± 0.88 ^b^	3.59 ± 0.74 ^b^
Food conversion ratio (%)—FCR	2.32± 0.57 ^a^	1.50± 0.25 ^b^	1.41± 0.07 ^b^
Survival Rate (%)—SR	95.30	96.70	96.30

Values are mean ± SD of three replicates. Values with the different letters in the same row are significantly different (*n* = 15). (^a^, ^b^, and ^c^) in a row indicates that similar superscripts do not differ (*p* > 0.05), and different superscripts indicate a significant difference (*p* < 0.05). * FRT—fermented rooibos tea-supplemented diet; * GRT—green rooibos tea-supplemented diet; basal diet—non-supplemented diet.

## Data Availability

The raw data supporting the conclusions of this article will be made available by the authors without undue reservation.
